# Supplementary material for: Interpretation of network-based integration from multi-omics longitudinal data
Source: Nucleic Acids Res. 2021 Dec 9;50(5):e27. doi: 10.1093/nar/gkab1200 (PMC8934642; doi:10.1093/nar/gkab1200)
Supplement: gkab1200_Supplemental_Files [file gkab1200_supplemental_files.zip › netOmics-supp_v3.1.pdf]

Supplemental material: Interpretation of network-based integration from multi-omics longitudinal data

1 Supplementary Tables

Supplementary Table 1: HeLa cell cycling study: network statistics of **(A.)** ARACNe gene networks and sub-networks by kinetic clusters. **(B.)** Protein-Protein Interaction sub-networks from BioGRID interaction database with only measured molecules. **(C.)** Same as B. with the addition of BioGRID first degree proteins.

**(A.)**	Connected Nodes	Isolated Nodes	Edges
Cluster 1	223	10	2338
Cluster 2	67	14	224
Cluster 3	25	10	33
Cluster 4	86	11	884
Entire network	421	25	4079

**(B.)**	Connected Nodes	Isolated Nodes	Edges
Cluster 1	1,784	29	19,714
Cluster 2	20	33	15
Cluster 3	9	41	6
Entire network	1,886	30	21,679

**(C.)**	Connected Nodes	Edges
Cluster 1	24,300	1,177,556
Cluster 2	5,327	82,438
Cluster 3	4,805	131,256
Entire network	24,498	1,518,635

Supplementary Table 2: HeLa cell cycling study: Over Representation Analysis results. Significant GO terms (BP, MF, CC) enriched from mRNA, translation products and proteins list set separately. ORA was performed by kinetic clusters and with entire sets of molecules. Additionally, p-values of enriched terms shared between mRNA, translation products and proteins were combined using Fisher's combined probability test. External file : ‘table_hela_ora.xlsx‘

Supplementary Table 3: HeLa cell cycling study: Random Walk with restart results on multi-omics network. First sheet ("mechanism"): Top 25 closest nodes from GO terms seeds. Second sheet ("prediction"): Closest GO term node (BP, MF,CC) from unlabelled seeds. Third sheet ("cluster"): Top 10 closest nodes from seeds. Nodes were labelled according to their kinetic clusters. Results were filtered to show only seeds with different clusters from the other nodes. External file : ‘table_hela_rwr.xlsx‘

Supplementary Table 4: Dynamic response to maize aphid feeding study: network statistics of ARACNe gene networks and sub-networks by kinetic clusters.

	Cluster 1	Cluster 2	Cluster 3	Cluster 4	Entire Network
Connected Nodes	180	153	42	64	473
Isolated Nodes	334	357	48	72	777
Edges	204	206	45	61	659

Supplementary Table 5: Dynamic response to maize aphid feeding study: Over Representation Analysis results. Significant GO terms (BP, MF, CC) enriched from mRNA, and proteins list set separately. ORA was performed by kinetic clusters and with entire sets of molecules. External file : 'table_maize_ora.xlsx'

Supplementary Table 6: Dynamic response to maize aphid feeding study: Random walk with restart results on multi-omics network. First sheet ("mechanism"): Top 25 closest nodes from GO terms seeds. Second sheet ("prediction"): Closest GO term node (BP, MF,CC) from unlabelled seeds. Third sheet ("cluster"): Top 10 closest nodes from seeds. Nodes were labelled according to their kinetic clusters. Results were filtered to show only seeds with different clusters from the other nodes. External file : 'table_maize_rwr.xlsx'

Supplementary Table 7: Diabetes seasonal study: Enrichment analysis from RNA, cytokines and proteins. Sheets 1,2,3: Gene over representation analysis results for cluster 1, 2 and both clusters combined. Sheets 4,5,6: MedlineRanker gene-related disease enrichment analysis results for cluster 1, 2 and both clusters combined. External file : 'table_diabetes_enrichment.xlsx'
